# Occurrence and Biological Cost of *mcr-1*-Carrying Plasmids Co-harbouring Beta-Lactamase Resistance Genes in Zoonotic Pathogens from Intensive Animal Production

**DOI:** 10.3390/antibiotics11101356

**Published:** 2022-10-05

**Authors:** Tiago Lima, Dina Loureiro, Ana Henriques, Fernando Ramos, Constança Pomba, Sara Domingues, Gabriela Jorge da Silva

**Affiliations:** 1 Faculty of Pharmacy, University of Coimbra, 3000-458 Coimbra, Portugal; 2 Center for Neuroscience and Cell Biology, University Coimbra, 3004-517 Coimbra, Portugal; 3 ALS-Controlvet, 3460-070 Tondela, Portugal; 4 REQUIMTE/LAQV, R. D. Manuel II, 4051-401 Oporto, Portugal; 5 Centre for Interdisciplinary Research in Animal Health, Laboratory of Antibiotic Resistance, CIISA, Faculty of Veterinary Medicine, University of Lisbon, 1300-477 Lisbon, Portugal

**Keywords:** colistin resistance, *mcr-1*, CTX-M, fitness cost, conjugation, livestock

## Abstract

Colistin is classified as a high-priority critical antimicrobial by the World Health Organization (WHO). A better understanding of the biological cost imposed by *mcr*-plasmids is paramount to comprehending their spread and may facilitate the decision about the ban of colistin in livestock. This study aimed to assess the prevalence of *mcr* and ESBL genes from 98 *Escherichia coli* and 142 *Salmonella enterica* isolates from food-producing animals and the impact of the *mcr-1* acquisition on bacterial fitness. Only *mcr-1* was identified by multiplex PCR (*mcr-1* to *mcr*-*10*) in 15.3% of *E. coli*. Colistin MICs ranged between 8–32 mg/L. In four isolates, *bla*_*TEM-1*_, *bla*_*CTX-M-1*_, and *bla*_*CTX-M-15*_ co-existed with *mcr-1*. The IncH12, IncHI1, IncP, IncN, and IncI plasmids were transferred by conjugation to *E. coli* J53 at frequencies of 10^−7^ to 10^−2^ cells/recipient. Growth kinetics assays showed that transconjugants had a significantly lower growth rate than the recipient (*p* < 0.05), and transconjugants’ average growth rate was higher in the absence than in the presence of colistin (1.66 versus 1.32 (*p* = 0.0003)). Serial transfer assay during 10 days demonstrated that plasmid retention ranged from complete loss to full retention. Overall, *mcr-1*-bearing plasmids impose a fitness cost, but the loss of plasmids is highly variable, suggesting that other factors beyond colistin pressure regulate the plasmid maintenance in a bacterial population, and colistin withdrawal will not completely lead to a decrease of *mcr-1* levels.

## 1. Introduction

Extensive use and misuse of antimicrobial agents are recognised as the most important causes of the emergence and selection of antimicrobial resistance worldwide. Nowadays, antimicrobial resistance has been widely considered a global threat to human and animal health and should be seen from a One Health perspective [1,2].

In veterinary medicine and intensive animal farm production, the overuse of antibiotics with therapeutic, prophylactic, and metaphylatic purposes led to the emergence of important clinical resistance genes, such as extended-spectrum beta-lactamases (ESBLs) and mobilised colistin resistance (*mcr*) genes, in bacteria of animal origin that may enter in the food chain and/or spread into the environment [3]. There are several reports of the prophylactic administration of cephalosporins in intensive pig production to prevent neonatal infections, namely *Streptococcus suis* infections, navel infections, arthritis and colibacillosis diarrhoea [4]. Colistin is approved in pig production in some countries for the control of *Enterobacteriaceae* infections, particularly for those caused by *Escherichia coli* [5]. In addition, according to the European Medicines Agency report, colistin should only be used for therapy or metaphylaxis purposes in food-producing animals [6]. However, according to the recommendations for the prudent and responsible use of antibiotics used in animals, colistin should have its use restricted and, whenever possible, its use should be based on antimicrobial susceptibility tests, given its extreme importance in human medicine thus following a One Health approach [7].

The first report of ESBL-producing bacteria was in 1980, shortly after the introduction of the third-generation cephalosporins in clinical practice. Nowadays, there are several types of ESBL that promote resistance to all beta-lactams, except carbapenems. The most widespread are the ESBL types generated due to point mutations in the classical penicillinases SHV-1/2, TEM-1/2 and the CTX-M family beta-lactamases [8].

The lack of effective antibiotics in human medicine, particularly to fight carbapenemase-producing *Enterobacterales,* led to a renewed interest in the old antibiotic colistin, which became one of the last-resort therapeutic options [9]. Colistin, also known as polymyxin E, was used in human medicine several decades ago, but it was banned due to its neurotoxicity and nephrotoxicity. In contrast, it has been widely used in animal production practice in numerous countries for therapeutic, prophylactic and growth promotion purposes, mostly in pigs, poultry and cattle, due to its efficiency and low cost [10].

The prevalence of colistin resistance has been gradually reported in the last few years, and understanding the underlying resistance mechanisms is a priority [11]. Colistin resistance is mainly associated with LPS structure modifications, with consequent reduced or absent affinity to colistin [10]. Until 2015, all known colistin resistance mechanisms were chromosomally encoded as a result of specific mutations that led to an overexpression of LPS-modifying genes [12]. Then, the first plasmid-mediated colistin resistance gene (*mcr-1*) was identified in *E. coli* isolates from pigs’ samples, retail meat and human clinical isolates in China. The *mcr* gene encodes a phosphoethanolamine transferase that adds a phosphoethanolamine group to Lipid A, leading to decreased binding of colistin to LPS [13]. Since then, *mcr-1* homologs (*mcr-1* to *mcr-10*) and several variants have been identified worldwide in diverse Gram-negative bacteria of different origins, mainly associated with food-producing animals [10,14,15,16,17].

It is generally assumed that antibiotic resistance by the acquisition of mobile genetic elements, like plasmids, confers a reduction in the fitness of the recipient cell that is expressed as reduced growth rates, lower virulence, lower transmission rates or less invasiveness [18]. However, the fitness cost is strongly dependent on the plasmid backbone and on the host cell type [19]. In addition, plasmids may harbour diverse resistance genes, which under the selective pressure of antibiotic treatment, may result in co-selection of the plasmid-carrying multiple resistance genes [20].

The co-occurrence of ESBL and *mcr* genes has been reported [4,15,16]. Their dissemination is fuelled by horizontal gene transfer mechanisms, such as conjugation, which may spread to pathogenic Gram-negative bacteria, further limiting the treatment options [10,21]. Therefore, the surveillance and molecular characterization of colistin resistance in zoonotic pathogens play a key role in the need to decide on restrictions on antibiotic use in animal production [22]. Moreover, understanding the biological cost of the acquisition of *mcr*-carrying plasmids is important to infer their stability in the host and dissemination.

The aim of this study was to assess the prevalence of *mcr* and ESBLs genes in *E. coli* and *Salmonella enterica* isolates, two well-known zoonotic pathogens, collected from intensive farming animals and farm environments, to assess the potential of horizontal dissemination of these genes and to evaluate the impact of *mcr-1* acquisition in the fitness of the host.

## 2. Results

### 2.1. Origin of Bacterial Isolates and Antimicrobial Susceptibility Test

A total of 240 bacterial isolates were collected: 142 *S. enterica* and 98 *E. coli* from different sources (Table 1 and Table 2) and from diverse Portuguese and Spain regions. The majority of the isolates were from poultry (Table 1). *S. enterica* serovar Typhimurium was the most prevalent serotype detected, followed by the serovar Enteritidis in poultry. Sixty-eight (47.9%) *S. enterica* isolates were recovered from the farm environment and one from animal-derived food (Table 2).

Bacterial susceptibility testing showed that in *S. enterica,* 41 isolates (28.87%) were resistant to amoxicillin, 26 (18.31%) were resistant to tetracycline, 6 (4.23%) to the combination amoxicillin/clavulanic acid, 3 (2.11%) to cephalosporins, 3 (2.11%) to aztreonam, and 3 (2.11%) to quinolones. Regarding *E. coli*, it was found 77 (78.57%) tetracycline-resistant isolates, 72 (73.47%) amoxicillin-resistant isolates, 45 (45.92%) quinolone-resistant isolates, 13 (13.27%) resistant isolates to the combination amoxicillin/clavulanic acid, 6 (6.12%) aztreonam-resistant isolates and 3 (4.08%) resistant isolates to cephalosporins.

Phenotypic screening of ESBL suggested that 18 (12.68%) *S. enterica* and 14 (14.29%) *E. coli* were ESBL producers.

It was observed that 94 bacterial isolates (39.17%) grew in EMB agar supplemented with 3.5 mg/L of colistin, of which 37 grew after 24 h (30 *E. coli* and 7 *S. enterica*; 15.42%) and 57 (20 *E. coli* and 37 *S. enterica*) only after 48 h (23.75%) of incubation.

### 2.2. Screening of mcr and ESBL Genes

The multiplex PCR allowed for the detection of 15 *E. coli* (15.31%) carrying *mcr-1,* but not in *S. enterica* isolates. The other *mcr* genes were not detected in any isolate. All the *mcr-1* positive strains were classified as resistant to colistin, with MICs values ranging from 8 to 32 mg/L. Two of the *mcr-1* positive *E. coli* strains only grew after 48 h under colistin selection, despite the MIC values of 8 and 16 mg/L. The majority of the *mcr-1* positive strains (*n* = 9; 60%) were collected from pig samples, while the others were from poultry (*n* = 4; 27%) and rabbits (*n* = 2; 13%).

Phenotypic screening of ESBL revealed 13.33% (32/240 isolates) of positivity, and all were screened for *bla*_TEM_, *bla*_SHV_ and *bla*_CTX-M_ genes. Twenty beta-lactamase-producing isolates (8.33%) were found, including 16.33% *E. coli* (16/98 *E. coli*) and 2.82% *S. enterica* (4/142 *S. enterica*). The four *S. enterica* isolates carried *bla*_*TEM-1*_ (2.82%). In relation to the *E. coli* isolates, 10 (10.20%) carried only *bla*_*TEM-1*_. Five (5.10%) *E. coli* isolates carried, beyond *bla*_*TEM-1*_, *mcr-1* (*n* = 2)*, mcr-1 and bla*_*CTX-M-15*_ (*n* = 1), *bla*_*CTX-M-1*_ (*n* = 1) and *bla*_OXY-2_ (*n* = 1). One *E. coli* carried both *mcr-1* and *bla*_*CTX-M-1*_. These results are summarised in Table 3.

### 2.3. Conjugation Experiments

Conjugation assays were performed with the 15 *mcr-1 E. coli* positive strains and *E. coli* J53 as the recipient cell. The *mcr-1* gene was successfully transferred to *E. coli* J53 from all the donor cells at a frequency between 10^−7^ to 10^−2^ cells per recipient (Table 3). Additionally, *bla*_*TEM-1*_ and *bla*_*CTX-M-1*_ were also co-transferred in *E. coli* 166, *E. coli* 170, *E. coli* 186 and E. *coli* 189, but not the *bla*_*CTX-M-15*_ in *E. coli* 186 (Table 3). The success of the gene transfer was confirmed by PCR detection of *mcr-1* and beta-lactamase genes. The transconjugants showed a resistance profile to colistin identical to the donors, with MICs values identical to the *mcr-1*-borne plasmids donor strain. PCR-based replicon typing showed that these genes were located on IncH12, IncHI1, IncP IncN and IncI_1_ plasmids.

### 2.4. Growth Rates and Plasmid Stability

**Figure 1:**
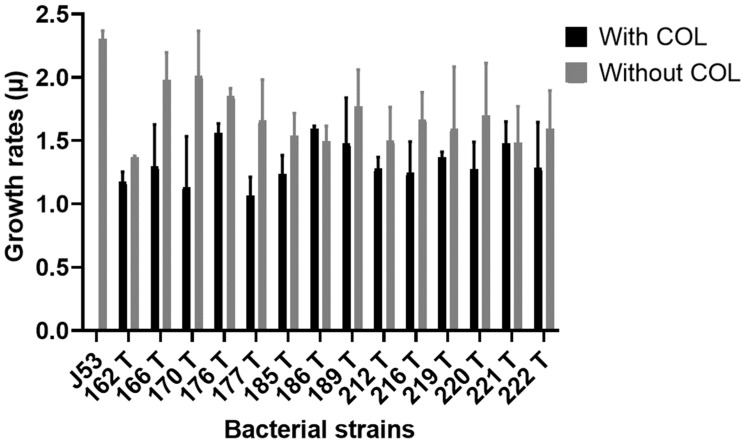
Growth kinetics assay results. Growth kinetics assays revealed that transconjugants have a mean growth rate of 1.66 in absence of COL (blue bars) and 1.32 in presence of COL (purple bars), *p*-value = 0.0003.

Growth kinetics of *E. coli* J53 and its *mcr-1*-carrying transconjugants were investigated. Significant growth rate differences between *E. coli* J53 and transconjugants 162T, 185T, 186T, 212T and 221T were found (*p* < 0.05). In addition, a significant difference in growth rates (*p* = 0.0003) was observed between transconjugants in the absence and in the presence of colistin, after 30 h assessment, with a mean growth rate of 1.66 and 1.32, respectively (Figure 1).

**Figure 2:**
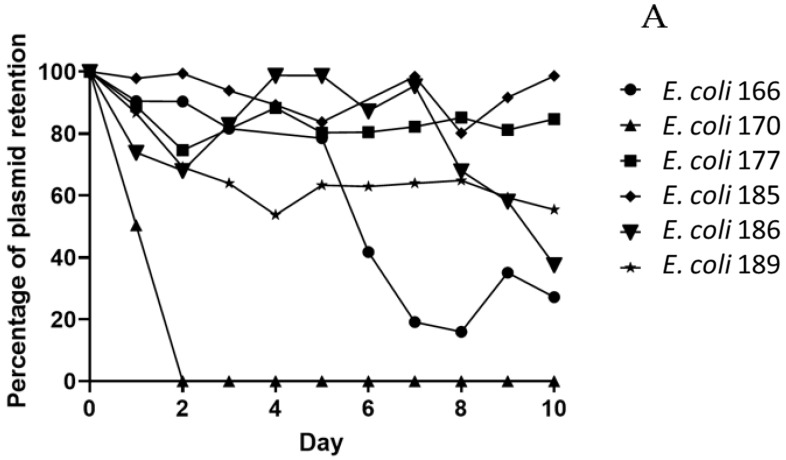

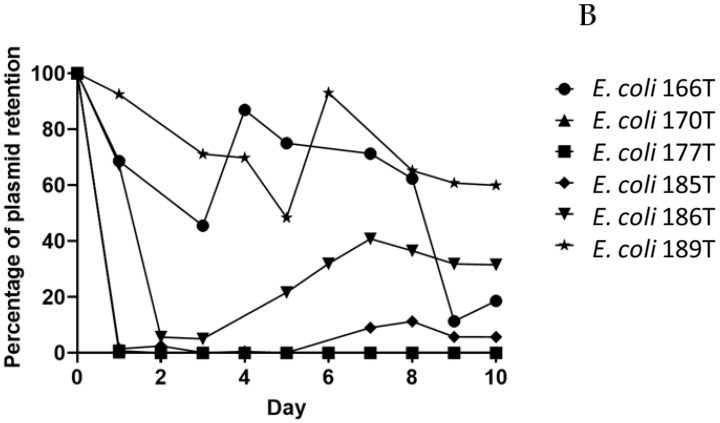
Stability of mcr-1 plasmids. Assessment of *E. coli* donor strains (**A**) and its transconjugants (**B**) over 10 days in the absence of colistin.

The stability of the *mcr-1* plasmids was determined by serial passages for 10 days in the absence of colistin. As shown in Figure 2A,B, plasmid retention rates were diverse. Briefly, in *E. coli* 177 and 185, IncHI2 plasmids were highly retained (>85%) but lost in their transconjugants (<95%). Otherwise, IncHI2 plasmids of *E. coli* 186 and its transconjugant were maintained in 37% and 31%, respectively. IncP plasmids were lost in both *E. coli* 170 and in its transconjugant; in contrast, in *E. coli* 166, 27% of parental strain and only 19% of transconjugants IncP plasmid was retained. Finally, an untypable plasmid was maintained stably in 60% of *E. coli* 189 and its transconjugants at least 10 days of passage in an antibiotic-free environment.

## 3. Discussion

In this study, *E. coli* and *S. enterica* isolates were recovered from animal biological and food samples and from farm environments in Portugal and Spain with the objective of assessing the epidemiology of *mcr-1* to *mcr-10* genes and ESBL genes.

Only the *mcr-1* gene was identified in *E. coli* samples. The majority (60%) of *mcr-1* identified in *E. coli* was from swine samples, which is in accordance with most of the current reports, where the main sources of *E. coli* carrying *mcr-1* are pigs, poultry samples and their derivate food products [16,23,24,25]. Yet, the majority of samples were from poultry, with the majority of isolates being *S. enterica*, including from boots’ swabs. Surprisingly, *mcr* genes were not detected in *S. enterica* despite the growth in media supplemented with colistin. Although less frequently than in *E. coli*, there are several reports worldwide of the occurrence of *mcr* genes in *S. enterica*, mostly the *mcr-1* gene, including in Portugal [10,25,26,27]. The presence of chromosomal mutations of the PmrA/PmrB and PhoP/PhoQ two-component regulatory systems, which are responsible for the biosynthesis of L-Ara4N and PEtn [10], or other uncharacterised variants of *mcr* genes may explain the growth of some *S. enterica* strains under colistin selection. The mechanisms and evolutionary pathways resulting in decreased susceptibility to colistin in certain *Salmonella* serovars remain to be fully understood, but bacterial tolerance, persistence or heteroresistance to antimicrobials and natural and spontaneous phenomena may be an explanation for bacterial growth in the presence of colistin [28,29].

Intensive swine production has been pointed to as the main source of emergence and spread of mobile colistin resistance due to the wide use of colistin for treatment and control of gastrointestinal infections caused by *Enterobacterales* in pigs and piglets [5,30]. Also, in industrial rabbit breeding, the recorded consumption of colistin is relevant, and in fact, we found the *mcr-1* gene in two *E. coli* isolates from rabbit meat, which is rarely reported [31,32,33]. It is highly expected that transmission between intensive farming animals is facilitated by close contact between them [34]. The food chain may play a major role in the spread of colistin resistance since *mcr* genes have been mostly found in samples from food-producing animals. There are several reports from human clinical samples [5]. The rate of *mcr* genes that have been found in our study and in the rest of Europe [35,36,37] was lower compared to the high rates observed in China. This may be explained by the previously high levels of colistin consumption observed in some Asian Countries, including China, where the use of colistin as a feed additive was allowed until recently as a growth promoter [37,38].

The co-occurrence of *mcr-1* with other clinically relevant resistance genes in *E. coli* was previously reported [4,15,16,24,39]. Here, the strains showed high levels of resistance to tetracycline and beta-lactams, as expected, because these antimicrobial agents were and are widely used in livestock production to prevent and control respiratory and gastrointestinal disease [40], including in Portugal [41]. Although ciprofloxacin is not used in livestock production, some isolates were ciprofloxacin-resistant. This can be explained by the cross-resistance to enrofloxacin, which is commonly used in farm animals and is partially metabolised to ciprofloxacin in animals [42]. Moreover, the *mcr-1* gene can easily spread due to the use of colistin or co-selection with other antibiotic classes [6]. Thus, the administration of different antibiotics to animals plays a key role in the development and spread of various resistance profiles [43].

Phenotypic screening of ESBL indicated a higher production of these enzymes compared to the PCR detection, which suggests that other less common ESBLs than *bla*_*CTX-M-1*_ and *bla*_*CTM-M-15*_ that were identified in this study may be produced. Additionally, there seems to be a connection between *mcr-1* and ESBL genes, which can lead to co-selection of the resistance to beta-lactams and colistin.

All *mcr-1*-carrying *E. coli* isolates were able to transfer the colistin resistance gene by conjugation at different frequencies, as reported [35]. The *mcr-1* gene has been often found on IncHI2, IncHI1, IncP, IncI1and IncN conjugative plasmids [30,44]. Other reports indicate that IncX4 and IncHI2 are the two major plasmid incompatibility groups implicated in *mcr-1* dissemination [27]. The genetic platforms of *mcr* in this study were variable. The plasmid IncHI2 was not completely lost in the absence of colistin, which might explain the highest frequency found. Moreover, it was shown that the dissemination of *mcr-1* is linked with the co-spread of ESBLs, namely *bla*_*CTX-M-1*_. The MICs for colistin for transconjugants showed a similar value to the donor strain, suggesting that colistin resistance is specific and not influenced by other resistance mechanisms in the donor strains. Nevertheless, the colistin-resistant wild strains may display chromosomal resistance genes.

The dissemination and the high diversity of *mcr-*encoding plasmid backbones assume greater relevance in the context of livestock production, particularly in swine production [5,21,45,46]. Animal manure should be regarded as a source of antibiotic residues and DNA, and in this case, it is not clear that the processing through composting is effective in reducing amounts of colistin or *mcr* genes in pig manure [5,21,46]. The role of animal manure used in land fertilization and in the environmental dissemination of *mcr* genes should be further studied.

Many isolates, especially from *S. enterica*, grew with colistin only after 48 h of incubation. As observed for two *E. coli* isolates, our study demonstrated that the prevalence of *mcr* genes might be underestimated because some positive *mcr-1* strains did not show visible growth after 24 h of incubation. Since *mcr-1* may be present in colistin-susceptible strains, it has been suggested that *mcr* genes may play a physiological role in bacterial cells [47]. Thus, the prevalence of these genes may be underestimated by the breakpoint value that might need an update and/or by the time of incubation, as shown in the present study.

In addition, the slow growth of some colistin-resistant isolates might be related to a fitness cost. Studying the biological cost of exogenous DNA in the recipient cell is important to comprehend the dynamic of transmission of plasmids in a bacterial population and to further understand whether colistin withdrawal in intensive animal production may attenuate the spread of the *mcr-1* gene with positive implications in public health in the view of the One Health context. A report showed that in China, after the ban of colistin as a growth promoter in 2017, the prevalence of colistin resistance and *mcr* genes was reduced [48]. However, this is not a paradigm [49,50]. To assess the biological cost of *mcr-*carrying-plasmid acquisition, we compared the growth rate of transconjugants and the recipient strain *E. coli* J53, observing that the carriage of the *mcr*-plasmid imposes a fitness cost to the host, as previously described [51,52,53]. Also, the comparison of growth rates between transconjugants in the presence and absence of colistin showed that the presence of colistin impairs the in vitro growth of *mcr-1*-carrying bacteria resulting in reduced fitness. This suggests that colistin withdrawal from intensive animal production could indeed attenuate the spread of the *mcr-1* gene because susceptible strains would be more fit. However, plasmid stability assays revealed that the loss of plasmids was highly variable, ranging from complete loss to full retention. IncHI2 was more retained than others, which might explain their higher prevalence association with *mcr* genes, while other plasmids, such as IncP, rarely described as carrying *mcr*-genes, were partially or completely lost [51,54]. These findings show that the acquisition of *mcr-1*-carrying plasmids reduces the fitness of the host, but plasmid loss in the absence of colistin is highly variable, and other factors beyond colistin pressure contribute to resistance-plasmid maintenance in a bacterial population. The variability in plasmid stability may be related to plasmid features (type, size) and bacterial host [51,55]. Compensatory adaptations may explain the *mcr-1* expression at a low fitness cost. However, the mechanisms that are responsible for the equilibrium between *mcr-1* expression to provide protection in the colistin environment and normal cellular functions remain unknown and should be further studied [50].

## 4. Materials and Methods

### 4.1. Bacterial Isolates

Ninety-eight *E. coli* and 142 *S. enterica* isolates were collected from animals of intensive farming production and farmer’s boots between January 2016 and April 2018 by ALS-Controlvet (Tondela, Portugal). These isolates were collected from swine, poultry, rabbit, sheep and bovine from Portugal and Spain. All *S. enterica* isolates were serotyped accordingly to the Kauffmann-White scheme [56].

### 4.2. Antimicrobial Susceptibility Testing

The antimicrobial susceptibility testing was performed by the disc diffusion method with the antibiotics: amoxicillin/clavulanic acid, ampicillin, apramycin, cefalexin, ceftiofur, doxycycline, erythromycin, enrofloxacin, florfenicol, flumequine, lincomycin/spectinomycin, nalidixic acid, neomycin, oxolinic acid, pipemidic acid, spiramycin, tetracycline, tiamulin, tilmicosin and trimethoprim/sulfamethoxazole (Oxoid, Basingstoke, UK), according to the European Committee on Antimicrobial Susceptibility Testing guidelines (EUCAST) [57]. Phenotypic detection of ESBL was performed by the double disc diffusion method with amoxicillin/clavulanic acid, aztreonam, ceftazidime and cefotaxime disks (Oxoid, Basingstoke, UK). The data were interpreted according to the EUCAST guidelines [57].

Screening of colistin-resistant isolates was performed according to a previously described method, with some modifications [58]. The SuperPolymyxin^®^ medium was developed for this purpose. However, our study was performed only with Gram-negative bacteria, and so neither amphotericin B nor daptomycin was used. Therefore, bacterial growth was observed in EMB agar (Liofilchem, Roseto degli Abruzzi, Italy) supplemented with 3.5 mg/L of colistin (Thermo Fisher Scientific, Waltham, MA, USA) after 24 and 48 h of incubation. Colistin susceptibility was evaluated by the broth microdilution method, according to EUCAST recommendations [57], determining the minimal inhibitory concentrations (MICs) both for wild strains and transconjugants.

### 4.3. Detection of Antimicrobial Resistance Genes

Screening of *bla*_SHV_, *bla*_TEM_ and *bla*_CTX-M_ type genes was performed by PCR with specific primers, as previously reported [59]. Bacterial isolates that grew in colistin-supplemented EMB agar were screened for *mcr-1* to *mcr-10* genes. PCR screening for *mcr-1* to *-5* was carried out using the multiplex PCR protocol, according to Rebelo et al., with some alterations [60]. Briefly, the multiplex PCR was divided into two: one for screening *mcr-1* and *mcr-3* and the second for *mcr-2*, *mcr-4* and *mcr-5*. Screening of *mcr-6* to *-9* genes was performed according to Borowiak et al. [61], and *mcr-10* was screened by simplex PCR using the in-house designed primers: *mcr-10*_fw [5′-ATTCCGTTTGTGCTGGTTGC-3′] and *mcr-10*_rv [5′-AACATACAGGGCACCGAGAC-3′] and the following conditions: initial denaturation at 95 °C for 60 s, followed by 30 cycles of denaturation at 95 °C for 30 s, annealing at 58 °C for 30 s and elongation at 72 °C for 60 s, and a final cycle of elongation at 72 °C for 10 min. The *mcr-10* amplicon size was 707 base pairs.

Identification of the resistance genes was confirmed by nucleotide sequencing of the amplicons (Stabvida, Caparica, Portugal).

### 4.4. Conjugation Assay and Plasmid Replicon Typing

Conjugation experiments were performed to determine the transferability of *mcr* genes using sodium azide-resistant *E. coli* J53 as a recipient strain at a bacterial cell ratio 1:10. Transconjugants were selected on Mueller Hinton (MH) Agar (Liofilchem, Roseto degli Abruzzi, Italy) plates supplemented with sodium azide (150 mg/L; Scharlab, Barcelona, Spain) and colistin (2 mg/L). The success of conjugation was further confirmed by *mcr-1*-PCR detection and colistin susceptibility testing by the microdilution method in transconjugants. Conjugation frequency (CF) was determined as the number of transconjugants per recipient cell.

Incompatibility groups were identified among *mcr-1*-borne plasmids carried by transconjugants by PCR-based replicon typing, using eighteen pairs of primers, as previously described [62].

### 4.5. Determination of Growth Rates

Growth rates of *E. coli* J53 and 14 transconjugants carrying the *mcr-1*-bearing IncH12, IncHI1, IncP IncN, IncI_1_, FIB plasmids and *bla*_*TEM-1*_, *bla*_*CTX-M-1*_ and *bla*_*CTX-M-15*_ genes were evaluated by measuring the optical density (OD) at 600 nm. Bacterial strains were inoculated in antibiotic-free and in 4 mg/L colistin-containing Luria-Bertani (LB) broth (Liofilchem, Roseto degli Abruzzi, Italy) at a 1:100 ratio. Bacterial growth was recorded by monitoring OD_600_ every 30 min for seven h and then at 8, 9, 10, 12, 24 and 30 h. The kinetics is expressed in a specific growth rate constant (µ).

Statistical analysis was performed using GraphPad Prism 8 software (GraphPad San Diego, CA, USA). The differences were assessed using paired, two-tailed *t*-test and ANOVA test. *p*-values < 0.05 were considered statistically significant.

### 4.6. Plasmid Stability Assay

The stability of *mcr-1*-bourne plasmids was investigated in vitro, as previously described [63]. Briefly, 10 µL of overnight cultures of six transconjugants and their donors were inoculated in 10 mL of antibiotic-free LB broth (1:1000 ratio). Sub-culturing was performed after 24 h of incubation at 37 °C in an orbital shaker at 120 rpm and repeated for 10 consecutive days. Each day, the culture broths were serially diluted and plated both onto antibiotic-free and 4 mg/L colistin-containing MH agar once 8 mg/L was the lowest MIC value found among these strains.

The percentage of plasmid retention was calculated by dividing the number of colonies on colistin-containing MH agar by the number of colonies on antibiotic-free MH agar.

## 5. Conclusions

We demonstrate that the prevalence of *mcr* genes might be underestimated due to the slow growth of colistin-resistant bacteria in phenotypic screening, suggesting the re-evaluation of reliable guidelines for epidemiological purposes. Colistin withdrawal from intensive farm production will not completely lead to a decrease of *mcr-1* levels since the reversal of colistin resistance mediated by *mcr* genes is not straightforward.

The results highlight the need of continuous surveillance of foodborne pathogens and implementation or improvement of antibiotic stewardship in animal production since it promotes the emergence of bacteria carrying important clinical resistance genes, which can enter the food chain and human gut. These data are important because they can provide a basis for the development of national policies, and they can help guide the risk of colistin resistance management and assess the effect on animal, environmental and public health of possible interventions following a One Health perspective. For efficient monitoring systems at the national level, coordination between the many stakeholders is essential.

With the application of sufficient science-based risk management policies that adhere to transdisciplinary recommendations, the One Health concept is more crucial than ever to better control colistin resistance at the interface between the human, animal and environment with the goal of achieving a balance between the need to protect public health and the potential impact of risk management measures on animal health.

## Figures and Tables

**Table 1:** Origin of *Escherichia coli* and serovar of *Salmonella enterica* isolates. The numbers at the end of table are the number of strains.

Source	*E. coli* (*n* = 98)	*S. enterica* (*n* = 142)	
Poultry		*S.* Typhimurium	19
		*S.* Enteritidis	14
		*S.* Anatum	11
		*S.* Infantis	11
		*S.* Havana	10
		*S.* 4,12:i:-	9
		*S.* Brandenburg	7
		*S.* Typhimurium -like	6
		*S.* Cerro	4
		*S.* Indiana	4
		*S.* Lexington	4
		*S.* Bredeney	3
		*S.* Rissen	3
		*S.* Virchow	3
		*S.* Kentucky	2
		*S.* Mbandaka	2
		*S.* Tennessee	2
		*S.* 9,46:-:-	1
		*S*. 3,15:z10:	1
		*S.* Salamae 4(5),12:b-	1
		*S.* Agona	1
		*S.* Bardo	1
		*S.* Berta	1
		*S.* Hadar	1
		*S.* Kottbus	1
		*S.* Llandoff	1
		*S.* Newport	1
		*S.* Ohio	1
		*S.* Seftenberg	1
		*S.* Schwarzengrund	1
		Not typed	6
	77		133
Swine		*S.* Derby	2
		*S.* Typhimurium	1
		Not typed	4
	16		7
Rabbit	3	-	-
Sheep	2	-	-
Animal feed	-	Not typed	1
Bovine	-	*S.* Anatum	1

**Table 2:** Frequency of *Escherichia coli* and *Salmonella enterica* according to biological, food and environmental samples.

	*E. coli* (*n* = 98)	*S. enterica* (*n* = 142)
Boots swabs		58
Carcasses/fresh meat	42	20
Ready to cook meat		18
Organs	40	2
Rectal swabs	6	
Faeces	6	24
Incubator/bed swabs	2	10
Neck skin		5
Abscess	2	
Frozen meat		2
Eggs		2
Animal feed		1

**Table 3:** Origin, phenotypic and genetic characteristics of antimicrobial resistance of *mcr-1* positive and/or ESBL-producing *E. coli* isolates.

Isolate	Source of Isolates	Date of Isolation	Growth in Agar ^a^ (h)	Resistance Genes	Colistin MIC ^b^ (mg/L)	Antimicrobial Resistance ^c^	Conjugation Frequency (Cells per Recipient)	*mcr-1* Plasmid Typing
***E. coli* 162**	Chicken organs	12/2017	24	*mcr-1*	8	AMC; AML; TE; CIP	10^−2^	P
***E. coli* 166**	Fowl carcasse	01/2018	24	*mcr-1*; *bla_TEM-1_*	16	AMC; AML; TE; CIP	10^−4^	HI2; P
***E. coli* 170**	Turkey carcasse	10/2017	24	*mcr-1*; *bla_TEM-1_*	8	AMC; AML; TE; CIP	10^−2^	P
***E. coli* 176**	Rabbit organs	11/2017	24	*mcr-1*	16	AML; TE; CIP	10^−6^	I1/Iγ
***E. coli* 177**	Rabbit organs	11/2017	24	*mcr-1*	32	AML; TE; CIP	10^−7^	HI2
***E. coli 182***	Chicken organs	11/2017	No growth	*bla_TEM-1_*; *bla_CTX-M-1_*	N.A.	ATM; CTX; AML; TE; CIP	n.a.	n.a.
***E. coli* 185**	Swine organs	11/2017	24	*mcr-1*	16	AML; TE; CIP	10^−7^	HI2
***E. coli* 186**	Swine organs	11/2017	48	*mcr-1*; *bla_TEM-1_*; *bla_CTX-M-15_*	16	ATM; CTX; AML; TE	10^−5^	HI2
***E. coli* 189**	Swine organs	11/2017	24	*mcr-1*; *bla_CTX-M-1_*	32	ATM; CTX; AML; TE; CIP	10^−4^	F (FIB)
***E. coli* 206**	Turkey organs	11/2017	48	*bla_TEM-1_*; *bla_OXY-2_*	N.A.	AMC, AML; TE; CIP; CEX	n.a.	n.a.
***E. coli* 212**	Swine rectal swab	12/2017	24	*mcr-1*	16	AML; TE; CIP	10^−3^	n.d.
***E. coli* 216**	Swine rectal swab	12/2017	24	*mcr-1*	16	AMC; AML; TE; CIP	10^−4^	N
***E. coli* 219**	Piglet faeces	12/2017	24	*mcr-1*	16	AML; TE	10^−2^	N
***E. coli* 220**	Piglet faeces	12/2017	24	*mcr-1*	8	AML; TE	10^−2^	HI1
***E. coli* 221**	Swine rectal swab	12/2017	24	*mcr-1*	16	AML; TE; CIP	10^−3^	HI1
***E. coli* 222**	Swine rectal swab	12/2017	48	*mcr-1*	8	AML; TE	10^−2^	n.d.
***E. coli* 226**	Poultry carcass	01/2018	24	*mcr-1*	16	AML; TE; CIP	10^−3^	n.d.

^a^ EMB supplemented with 3.5 mg/L colistin; ^b^ MICs for donors and transconjugants; ^c^ Abbreviations: AMC—Amoxicillin/Clavulanic acid; AML—Amoxicillin; TE—Tetracycline; CIP—Ciprofloxacin; ATM—Aztreonam; CTX—Cefotaxime; CEX—Cefalexin; n.a.—not applicable; n.d.—not determined.

## Data Availability

Not applicable.

## References

[1] WHO Global Action Plan on Antimicrobial Resistance. https://www.who.int/antimicrobial-resistance/publications/global-action-plan/en/.

[2] CDC Antibiotic Resistance Threats in the United States. https://www.cdc.gov/drugresistance/pdf/threats-report/2019-ar-threats-report-508.pdf.

[3] Economou V., Gousia P. (2015). Agriculture and food animals as a source of antimicrobial-resistant bacteria. Infect. Drug Resist..

[4] Lima T., Fernandes L., Matias M., Mateus A., Silveira E., Domingues S., Pomba C., Da Silva G.J. (2022). Longitudinal Study Detects the Co-Carriage of ESBL and *mcr-1* and *-4* Genes in *Escherichia coli* Strains in a Portuguese Farrow-to-Finish Swine Herd. Animals.

[5] Rhouma M., Beaudry F., Theriault W., Letellier A. (2016). Colistin in Pig Production: Chemistry, Mechanism of Antibacterial Action, Microbial Resistance Emergence, and One Health Perspectives. Front. Microbiol..

[6] EMA (2016). Updated Advice on the Use of Colistin Products in Animals within the European Union: Development of Resistance and Possible Impact on Human and Animal Health—EMA/CVMP/CHMP/231573/2016.

[7] European Medicines Agency (2019). Categorisation of Antibiotics in the European Union.

[8] Rawat D., Nair D. (2010). Extended-spectrum beta-lactamases in Gram Negative Bacteria. J. Glob. Infect. Dis..

[9] WHO Critically Important Antimicrobials for Human Medicine—3rd Rev. https://apps.who.int/iris/bitstream/handle/10665/77376/;jsessionid=0C947CA333F9F82CF7708F537AE6FB85?sequence=1.

[10] Lima T., Domingues S., Da Silva G.J. (2019). Plasmid-Mediated Colistin Resistance in *Salmonella enterica:* A Review. Microorganisms.

[11] Zafer M.M., El-Mahallawy H.A., Abdulhak A., Amin M.A., Al-Agamy M.H., Radwan H.H. (2019). Emergence of colistin resistance in multidrug-resistant *Klebsiella pneumoniae* and *Escherichia coli* strains isolated from cancer patients. Ann. Clin. Microbiol. Antimicrob..

[12] Pragasam A.K., Shankar C., Veeraraghavan B., Biswas I., Nabarro L.E., Inbanathan F.Y., George B., Verghese S. (2016). Molecular Mechanisms of Colistin Resistance in *Klebsiella pneumoniae* Causing Bacteremia from India-A First Report. Front. Microbiol..

[13] Liu Y.Y., Wang Y., Walsh T.R., Yi L.X., Zhang R., Spencer J., Doi Y., Tian G., Dong B., Huang X. (2016). Emergence of plasmid-mediated colistin resistance mechanism MCR-1 in animals and human beings in China: A microbiological and molecular biological study. Lancet. Infect. Dis..

[14] Hadjadj L., Baron S.A., Olaitan A.O., Morand S., Rolain J.M. (2019). Co-occurrence of Variants of *mcr-3* and *mcr-8* Genes in a *Klebsiella pneumoniae* Isolate From Laos. Front. Microbiol..

[15] Fournier C., Aires-de-Sousa M., Nordmann P., Poirel L. (2019). Occurrence of CTX-M-15- and MCR-1-producing Enterobacterales in pigs in Portugal: Evidence of direct links with antibiotic selective pressure. Int. J. Antimicrob. Agents.

[16] Manageiro V., Clemente L., Romao R., Silva C., Vieira L., Ferreira E., Canica M. (2019). IncX4 plasmid carrying the new *mcr-1.9* gene variant in a CTX-M-8-producing *Escherichia coli* isolate recovered from swine. Front. Microbiol..

[17] Wang C., Feng Y., Liu L., Wei L., Kang M., Zong Z. (2020). Identification of novel mobile colistin resistance gene *mcr-10*. Emerg. Microbes Infect..

[18] Andersson D.I. (2006). The biological cost of mutational antibiotic resistance: Any practical conclusions?. Curr. Opin. Microbiol..

[19] Humphrey B., Thomson N.R., Thomas C.M., Brooks K., Sanders M., Delsol A.A., Roe J.M., Bennett P.M., Enne V.I. (2012). Fitness of *Escherichia coli* strains carrying expressed and partially silent IncN and IncP1 plasmids. BMC Microbiol..

[20] Andersson D.I., Hughes D. (2010). Antibiotic resistance and its cost: Is it possible to reverse resistance?. Nat. Rev. Microbiol..

[21] Lima T., Domingues S., Da Silva G.J. (2020). Manure as a Potential Hotspot for Antibiotic Resistance Dissemination by Horizontal Gene Transfer Events. Vet. Sci..

[22] Liu Y., Liu J.H. (2018). Monitoring colistin resistance in food animals, an urgent threat. Expert Rev. Anti-Infect. Ther..

[23] Wang Y., Hu Y., Cao J., Bi Y., Lv N., Liu F., Liang S., Shi Y., Jiao X., Gao G.F. (2019). Antibiotic resistance gene reservoir in live poultry markets. J. Infect..

[24] Clemente L., Manageiro V., Correia I., Amaro A., Albuquerque T., Themudo P., Ferreira E., Canica M. (2019). Revealing *mcr-1*-positive ESBL-producing *Escherichia coli* strains among *Enterobacteriaceae* from food-producing animals (bovine, swine and poultry) and meat (bovine and swine), Portugal, 2010–2015. Int. J. Food Microbiol..

[25] Ling Z., Yin W., Shen Z., Wang Y., Shen J., Walsh T.R. (2020). Epidemiology of mobile colistin resistance genes *mcr-1* to *mcr-9*. J. Antimicrob. Chemother..

[26] Figueiredo R., Card R.M., Nunez J., Pomba C., Mendonca N., Anjum M.F., Da Silva G.J. (2016). Detection of an *mcr-1*-encoding plasmid mediating colistin resistance in *Salmonella enterica* from retail meat in Portugal. J. Antimicrob. Chemother..

[27] Campos J., Cristino L., Peixe L., Antunes P. (2016). MCR-1 in multidrug-resistant and copper-tolerant clinically relevant *Salmonella* 1,4,[5],12:i:-and *S.* Rissen clones in Portugal, 2011 to 2015. Euro Surveill. Bull. Eur. Sur Les Mal. Transm. = Eur. Commun. Dis. Bull..

[28] Band V.I., Weiss D.S. (2019). Heteroresistance: A cause of unexplained antibiotic treatment failure?. PLoS Pathog..

[29] Brauner A., Fridman O., Gefen O., Balaban N.Q. (2016). Distinguishing between resistance, tolerance and persistence to antibiotic treatment. Nat. Rev. Microbiol..

[30] Kieffer N., Aires-de-Sousa M., Nordmann P., Poirel L. (2017). High Rate of MCR-1-Producing *Escherichia coli* and *Klebsiella pneumoniae* among Pigs, Portugal. Emerg. Infect. Dis..

[31] Freitas-Silva J., Inacio A.S., Mourao J., Antunes P., Mendes A., de Carvalho A.P., Vasconcelos V., Peixe L., da Costa P.M. (2018). Occurrence of *mcr-1* in *Escherichia coli* from rabbits of intensive farming. Vet. Microbiol..

[32] Agnoletti F., Brunetta R., Bano L., Drigo I., Mazzolini E. (2018). Longitudinal study on antimicrobial consumption and resistance in rabbit farming. Int. J. Antimicrob. Agents.

[33] Wang X., Zhai Z., Zhao X., Zhang H., Jiang H., Wang X., Wang H., Chang W. (2021). Occurrence and characteristics of *Escherichia coli mcr-1*-like in rabbits in Shandong, China. Vet. Med. Sci..

[34] Argudin M.A., Deplano A., Meghraoui A., Dodemont M., Heinrichs A., Denis O., Nonhoff C., Roisin S. (2017). Bacteria from Animals as a Pool of Antimicrobial Resistance Genes. Antibiotics.

[35] Wang R., van Dorp L., Shaw L.P., Bradley P., Wang Q., Wang X., Jin L., Zhang Q., Liu Y., Rieux A. (2018). The global distribution and spread of the mobilized colistin resistance gene *mcr-1*. Nat. Commun..

[36] Tong H., Liu J., Yao X., Jia H., Wei J., Shao D., Liu K., Qiu Y., Ma Z., Li B. (2018). High carriage rate of *mcr-1* and antimicrobial resistance profiles of *mcr-1*-positive *Escherichia coli* isolates in swine faecal samples collected from eighteen provinces in China. Vet. Microbiol..

[37] Catry B., Cavaleri M., Baptiste K., Grave K., Grein K., Holm A., Jukes H., Liebana E., Lopez Navas A., Mackay D. (2015). Use of colistin-containing products within the European Union and European Economic Area (EU/EEA): Development of resistance in animals and possible impact on human and animal health. Int. J. Antimicrob. Agents.

[38] Sun J., Zhang H., Liu Y.H., Feng Y. (2018). Towards Understanding MCR-like Colistin Resistance. Trends Microbiol..

[39] Li B., Ke B., Zhao X., Guo Y., Wang W., Wang X., Zhu H. (2018). Antimicrobial Resistance Profile of *mcr-1* Positive Clinical Isolates of *Escherichia coli* in China From 2013 to 2016. Front. Microbiol..

[40] Burow E., Rostalski A., Harlizius J., Gangl A., Simoneit C., Grobbel M., Kollas C., Tenhagen B.A., Kasbohrer A. (2019). Antibiotic resistance in *Escherichia coli* from pigs from birth to slaughter and its association with antibiotic treatment. Prev. Vet. Med..

[41] Figueiredo R., Henriques A., Sereno R., Mendonca N., da Silva G.J. (2015). Antimicrobial resistance and extended-spectrum beta-lactamases of *Salmonella enterica* serotypes isolated from livestock and processed food in Portugal: An update. Foodborne Pathog. Dis..

[42] Schulz J., Kemper N., Hartung J., Janusch F., Mohring S.A.I., Hamscher G. (2019). Analysis of fluoroquinolones in dusts from intensive livestock farming and the co-occurrence of fluoroquinolone-resistant *Escherichia coli*. Sci. Rep..

[43] Huang X., Yu L., Chen X., Zhi C., Yao X., Liu Y., Wu S., Guo Z., Yi L., Zeng Z. (2017). High Prevalence of Colistin Resistance and *mcr-1* Gene in *Escherichia coli* Isolated from Food Animals in China. Front. Microbiol..

[44] Li X.P., Sun R.Y., Song J.Q., Fang L.X., Zhang R.M., Lian X.L., Liao X.P., Liu Y.H., Lin J., Sun J. (2020). Within-host heterogeneity and flexibility of *mcr-1* transmission in chicken gut. Int. J. Antimicrob. Agents.

[45] Brauer A., Telling K., Laht M., Kalmus P., Lutsar I., Remm M., Kisand V., Tenson T. (2016). Plasmid with Colistin Resistance Gene *mcr-1* in Extended-Spectrum-beta-Lactamase-Producing *Escherichia coli* Strains Isolated from Pig Slurry in Estonia. Antimicrob. Agents Chemother..

[46] Gao Y., Lu C., Shen D., Liu J., Ma Z., Yang B., Ling W., Waigi M.G. (2019). Elimination of the risks of colistin resistance gene (*mcr-1*) in livestock manure during composting. Environ. Int..

[47] Carroll L.M., Gaballa A., Guldimann C., Sullivan G., Henderson L.O., Wiedmann M. (2019). Identification of Novel Mobilized Colistin Resistance Gene *mcr-9* in a Multidrug-Resistant, Colistin-Susceptible *Salmonella enterica* Serotype Typhimurium Isolate. MBio.

[48] Wang Y., Xu C., Zhang R., Chen Y., Shen Y., Hu F., Liu D., Lu J., Guo Y., Xia X. (2020). Changes in colistin resistance and *mcr-1* abundance in *Escherichia coli* of animal and human origins following the ban of colistin-positive additives in China: An epidemiological comparative study. Lancet Infect. Dis..

[49] Kyung-Hyo D., Jae-Won B., Wan-Kyu L. (2020). Antimicrobial Resistance Profiles of *Escherichia coli* from Diarrheic Weaned Piglets after the Ban on Antibiotic Growth Promoters in Feed. Antibiotics.

[50] Yang Q., Li M., Spiller O.B., Andrey D.O., Hinchliffe P., Li H., MacLean C., Niumsup P., Powell L., Pritchard M. (2017). Balancing *mcr-1* expression and bacterial survival is a delicate equilibrium between essential cellular defence mechanisms. Nat. Commun..

[51] Ma K., Feng Y., Zong Z. (2018). Fitness cost of a *mcr-1*-carrying IncHI2 plasmid. PLoS ONE.

[52] Wu J., Dong X., Zhang L., Lin Y., Yang K. (2021). Reversing Antibiotic Resistance Caused by Mobile Resistance Genes of High Fitness Cost. mSphere.

[53] Li W., Liu Z., Yin W., Yang L., Qiao L., Song S., Ling Z., Zheng R., Wu C., Wang Y. (2021). MCR Expression Conferring Varied Fitness Costs on Host Bacteria and Affecting Bacteria Virulence. Antibiotics.

[54] Shen C., Zhong L.L., Yang Y., Doi Y., Paterson D.L., Stoesser N., Ma F., El-Sayed Ahmed M.A.E., Feng S., Huang S. (2020). Dynamics of *mcr-1* prevalence and *mcr-1*-positive *Escherichia coli* after the cessation of colistin use as a feed additive for animals in China: A prospective cross-sectional and whole genome sequencing-based molecular epidemiological study. Lancet Microbe.

[55] Wu R., Yi L.X., Yu L.F., Wang J., Liu Y., Chen X., Lv L., Yang J., Liu J.H. (2018). Fitness Advantage of *mcr-1*-Bearing IncI2 and IncX4 Plasmids in Vitro. Front. Microbiol..

[56] Grimont P.A., Weill F.X. (2007). Antigenic formulae of the *Salmonella* serovars. WHO Collab. Cent. Ref. Res. Salmonella.

[57] EUCAST (2019). Breakpoint Tables for Interpretation of MICs and Zone Diameters, Version 9.0. https://www.eucast.org/fileadmin/src/media/PDFs/EUCAST_files/Breakpoint_tables/v_9.0_Breakpoint_Tables.pdf.

[58] Nordmann P., Jayol A., Poirel L. (2016). A Universal Culture Medium for Screening Polymyxin-Resistant Gram-Negative Isolates. J. Clin. Microbiol..

[59] Mendonca N., Leitao J., Manageiro V., Ferreira E., Canica M. (2007). Spread of extended-spectrum beta-lactamase CTX-M-producing *Escherichia coli* clinical isolates in community and nosocomial environments in Portugal. Antimicrob. Agents Chemother..

[60] Rebelo A.R., Bortolaia V., Kjeldgaard J.S., Pedersen S.K., Leekitcharoenphon P., Hansen I.M., Guerra B., Malorny B., Borowiak M., Hammerl J.A. (2018). Multiplex PCR for detection of plasmid-mediated colistin resistance determinants, *mcr-1, mcr-2, mcr-3, mcr-4 and mcr-5* for surveillance purposes. Euro Surveill. Bull. Eur. Sur Les Mal. Transm. Eur. Commun. Dis. Bull..

[61] Borowiak M., Baumann B., Fischer J., Thomas K., Deneke C., Hammerl J.A., Szabo I., Malorny B. (2020). Development of a Novel *mcr-6* to *mcr-9* Multiplex PCR and Assessment of *mcr-1* to *mcr-9* Occurrence in Colistin-Resistant *Salmonella enterica* Isolates From Environment, Feed, Animals and Food (2011-2018) in Germany. Front. Microbiol..

[62] Carattoli A., Bertini A., Villa L., Falbo V., Hopkins K.L., Threlfall E.J. (2005). Identification of plasmids by PCR-based replicon typing. J. Microbiol. Methods.

[63] Bryksin A.V., Matsumura I. (2010). Rational design of a plasmid origin that replicates efficiently in both gram-positive and gram-negative bacteria. PLoS ONE.

